# Laparoscopic extralevator abdominoperineal resection for low rectal cancer: The myth of reinventing the wheel

**DOI:** 10.12669/pjms.40.1.7619

**Published:** 2024

**Authors:** Nighat Bakhtiar, Irfan-ul-Islam Nasir, Muhammad Fahd Shah, Ihtisham-Ulah, Osama Shakeel, Shahid Khattak, Aamir Ali Syed

**Affiliations:** 1 Nighat Bakhtiar Senior Instructor Surgical Oncology, Department of Surgical Oncology, Shaukat Khanum Memorial Cancer Hospital, And Research Centre (SKMCH&RC), Lahore, Pakistan; 2 Irfan-ul-Islam Nasir Consultant Colorectal Surgeon, Department of Surgical Oncology, Shaukat Khanum Memorial Cancer Hospital, And Research Centre (SKMCH&RC), Lahore, Pakistan; 3 Muhammad Fahd Shah Consultant Colorectal Surgeon, Department of Surgical Oncology, Shaukat Khanum Memorial Cancer Hospital, And Research Centre (SKMCH&RC), Lahore, Pakistan; 4 Ihtisham-Ulah Fellow Surgical Oncology, Department of Surgical Oncology, Shaukat Khanum Memorial Cancer Hospital, And Research Centre (SKMCH&RC), Lahore, Pakistan; 5 Osama Shakeel, Resident General Surgery, Department of Surgical Oncology, Shaukat Khanum Memorial Cancer Hospital, And Research Centre (SKMCH&RC), Lahore, Pakistan; 6 Shahid Khattak Consultant Surgical Oncologist, Department of Surgical Oncology, Shaukat Khanum Memorial Cancer Hospital, And Research Centre (SKMCH&RC), Lahore, Pakistan; 7 Aamir Ali Syed Consultant Surgical Oncologist, Department of Surgical Oncology, Shaukat Khanum Memorial Cancer Hospital, And Research Centre (SKMCH&RC), Lahore, Pakistan

**Keywords:** Oncological outcomes, Laparoscopic extralevator abdominoperineal excision, Low Rectal Cancer

## Abstract

**Background & Objective:**

To review oncological outcomes of laparoscopic extralevator abdominoperineal excision (LAP-ELAPE) for low rectal cancer.In locally advanced low rectal cancer, ELAPE which is en-bloc resection of levator muscles along with the tumor in a prone position has significantly decreased the rate of having either positive circumferential resection margin (CRM) or tumor perforation. The aim of the study was to determine the oncological outcomes of laparoscopic extralevator abdominoperineal excision (LAP-ELAPE) for low rectal cancer.

**Methods:**

This retrospective study was performed at Shaukat Khanum Cancer Hospital and Research Centre Lahore. Patients who underwent ELAPE for low rectal and anal cancer from January 2014 to December 2019 were selected. Data was collected using an electronic database through a hospital information system.

**Results:**

A total of 82 patients were included in the study having a median age of 39 years. Clinically preoperative tumor sizes were T2:2, T3:65, T4:15. Neo-adjuvant chemo radiotherapy was administered to 79 (96.3%) patients. Pathologically tumor sizes were T0:12, T2:15, T3:50, T4:5 with 79.2% (n=65) R0 resections. The mean operative time was 340.36±64.51 minutes and the mean blood loss was 99 milliliters. The mean postoperative hospital stay was 6.58±4.64 days. Seventeen (20.7%) cases had pathological circumferential resection margins positive (pCRM<1mm). However, tumor perforation was found in 8(9.8%) patients. Ninety days mortality was none while 36 patients experienced recurrence (local: 23, distant: 30, local + distant 17). The median survival time was 53.00±2.69 months.

**Conclusion:**

For locally advanced low rectal cancer, ELAPE has evolved as a safe oncological procedure with acceptable outcomes.

## INTRODUCTION

Colorectal cancer (CRC) is considered the third most common cancer in the world and has been classified as the second most important reason of cancer-related mortalities in 2018.^1^The surgical treatment for low rectal cancer is abdominoperineal excision (APE) in those cases where low anterior resection and anastomosis (LAR) is not possible.^2^ First, conventional abdominoperineal resection (cAPR) was performed by Miles.^3^ Later on, Heald coined the concept of total mesorectal excision in 1982, which has revolutionized the treatment of rectal cancer in the last few decades.^4^

cAPR is associated with two complications i.e., positive circumferential resection margin (CRM) and intra-operative tumor perforation (IOTP) which are estimated to be around 12-49% and 13.7-28.2% respectively.^5^-^7^ To overcome the increased rate of CRM positivity Holmes then introduced a new approach called “extralevator abdominoperineal excision” (ELAPE), which is en-bloc resection of levator muscles along with mesorectum in the prone position. This technique has resulted in a significantly decreased rate of complications like positive CRM and IOTP.^5^,^8^ A combination of neo-adjuvant treatment and ELAPE has contributed to achieving better survival outcomes in the last decade. Laparoscopic surgery for colonic cancer is now a well-known standard treatment for colonic cancers as compared to traditional open colectomy.^9^ However, its role in rectal cancer is somewhat controversial because of its efficacy regarding oncological safety. Nevertheless, many prospective studies have confirmed the safe and effective use of laparoscopy in the treatment of locally advanced low rectal carcinoma.^10^,^11^

In recent years, two non-inferiority studies raised questions on the safe use of laparoscopy in rectal carcinoma treatment, but the long-term results didn’t prove the initial claims.^12^,^13^ Many studies advocate the use of laparoscopic ELAPE (LAP-ELAPE) in low rectal cancer as a safer and better approach. There is no published literature from Pakistan regarding the use of LAP-ELAPE in low rectal cancer.^14^ This study aimed to look at the oncological outcomes of the locally advanced low rectal tumors treated with extralevator abdominoperineal resection.

## METHODS

### Patients

From January 2014 to December 2019, patients who underwent elective ELAPE for low rectal (<6 cm from the anal verge) and anal at SKMCH&RC, Pakistan were selected. It was a retrospective study from prospectively collected data from the hospital information system (HIS) with convenient sampling. Patients who were aged more than 18 years and of either sex with histology-proven anorectal cancer were included in the study. Patients who underwent cAPR were excluded. The demographics of the patients being treated at Shaukat Khanum Memorial Cancer Hospital and Research Center (SKMCH & RC) can be seen in this study.^15^

### Variables

Data was collected through the HIS electronic database of SKMCH&RC. Variables recorded were age, gender, pre-surgery histopathology, clinical staging, treatment received, type of surgery, and mode of surgery, pathological stage, intra-operative surgical parameters, post-operative morbidity, and mortality.

### Ethical Approval

Ethical approval was sought from the Institutional Review Board of SKMCH & RC (EX-03-04-20-03).

Preoperatively, all the patients underwent staging workup with digital rectal examination (DRE), colonoscopy, magnetic resonance imaging (MRI) of the pelvis, computed tomography (CT) scan of the chest and abdomen to rule out metastases of rectal cancer. All the patients were discussed in multidisciplinary team meetings and treatment intent was determined preoperatively depending on the staging workup. Almost all the patients received neo-adjuvant chemotherapy and radiotherapy. Post-treatment scans like an MRI pelvis and CT scan chest and abdomen were performed to re-stage the disease.

Surgery was performed between eight to ten weeks of completion of chemoradiotherapy. After recovery from surgery, depending on the pathology report of the tumor specimen, adjuvant treatment was offered. In the pathology specimen CRM was considered as positive when it was <1mm and negative when it was >1mm.^16^

Preoperative chemo-radiotherapy (CRT) was restricted to patients with T4 lesions or T3 with threatened/involved mesorectal fascia, or N1 or extra mesorectal involved lymph nodes on MRI. Tumors were identified as high, middle, or low based on proximity to the anal verge (10.1-15 cm; 5.1- 10 cm and < 5 cm, respectively) measured using MRI. Each surgery was performed laparoscopically as a standardized total mesorectal excision (TME) procedure.^17^

### Operative technique

**Fig.1:**
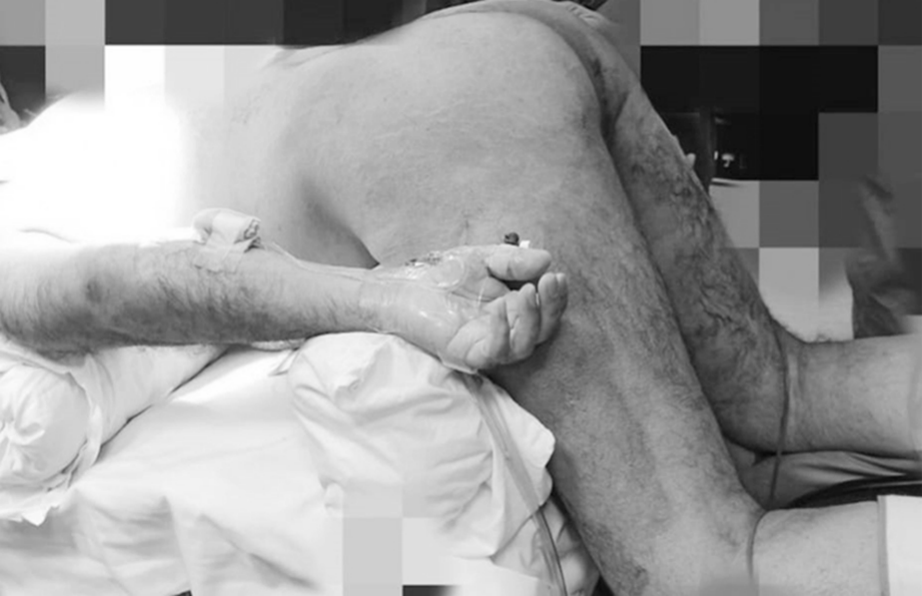
Prone Jack Knife position for the perineal part.

**Fig.2:**
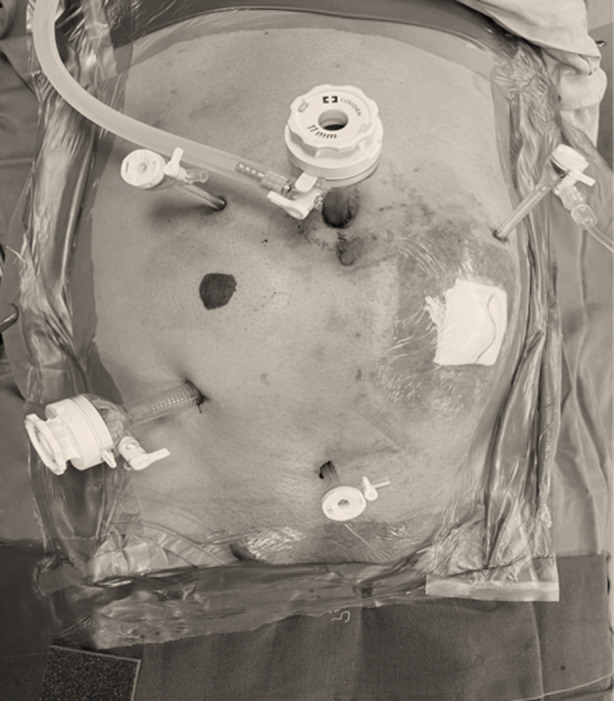
Position of Ports.

The patient was positioned in the Modified Loyd-Davis position for the abdominal part and in the Prone Jack Knife position for the perineal part (Fig.1) with different supportive devices used to prevent patient falls. Compression devices were used on the lower extremities. Prophylactic single-shot antibiotics were administered at the time of induction. Hasson technique was used for supra-umbilical optical port entry and further four more ports were inserted under direct vision as per Fig.2.

Trendelenburg position with right tilt of the table was made, so that the pelvis empties, the small bowel was moved towards the right side, and the sigmoid mesentery was exposed. Medial to lateral dissection performed with identification and preservation of left ureter and gonadal vessels. The inferior mesenteric artery is clipped at its origin sparing pre-aortic autonomic nerves. The inferior mesenteric vein is dissected, clipped, and transacted at the lower border of the pancreas to ensure oncological clearance. Lateral colonic mobilization ensures tension-free end stoma formation and the colon is prepared for transaction at a convenient point and is achieved with the help of an intra-corporal stapling gun. TME dissection is initiated by entering the avascular posterior TME plane using monopolar hook diathermy followed by development and dissection of the anterior, lateral, and posterior planes. A permanent stoma is fashioned in the left iliac fossa at the site which is marked pre-operatively.

**Fig.3:**
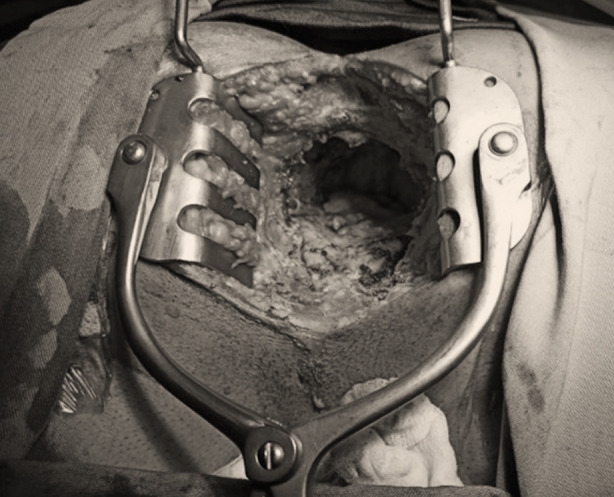
Perineal wound after removal of specimen.

The prone Jackknife position is made for the perineal part. Double purse-string suture is taken around the anus to avoid stool spillage and wound contamination. An elliptical incision is given and ischiorectal fat is dissected along with the Levator ani muscle. Circumferential dissection is performed, and the specimen is retrieved via the perineal wound (Fig.3). Omentum when bulky is mobilized and stitched in the pelvic cavity. Hemostasis is secured and washout is performed. The wound is closed in layers and a suction drain was placed in the perineal wound.

### Statistical analyses

Data was calculated using Statistical Package for the Social Sciences (SPSS 20) for Windows version 20 statistical software. Data was described using a median with minimum and maximum value for skew distributed quantitative variables. For categorical variables, a number of observations and percentages were reported. The study has complied with the SKMCH&RC guidelines on research involving human subjects.

## RESULTS

A total of 82 patients were included in the study. The median age of patients was 39 years ranging between 18 and 70 years. Out of 82 patients 62(75.6%) were males and 20(24.4%) were females. The mean Body mass index (BMI) of patients was 24.1±5.46. Neo-adjuvant chemoradiotherapy was given to 96.3% (n=79) of patients, in the remaining three patients, two were of malignant melanoma and one patient was offered upfront surgery. Other Preoperative patient demographics and tumor characteristics are displayed in Table-I. All patients underwent laparoscopic surgery and only one was converted from laparoscopic to open. The rest of the operative and in-patient details of patients are shown in Table-II.

**Table-I:** Patient demographics and Tumor Characteristics.

Variable	Frequency (n=82)	Percentage %
***Addiction***		
No Addiction	70	85.4
Smoking	1	1.2
Chewing Tobacco	11	13.4
***Histopathology***		
Adenocarcinoma	47	57.3
Signet ring cell carcinoma	22	26.8
Mucinous cell Carcinoma	11	13.4
Malignant Melanoma	2	2.4
***Grade***		
Well differentiated	16	19.5
Moderately Differentiated	25	30.5
Poorly differentiated	41	50
***ASA Grade***		
Grade 2	75	91.5
Grade 3	7	8.5
***Clinical Tumor Size***		
T2	2	2.4
T3	65	79.2
T4	15	18.3
***Clinical Nodal Status***		
N0	11	13.4
N1	34	41.5
N2	37	45.1
***Distant Metastasis***		
M0	79	96.3
M1	3	3.7
***Clinical Stage***		
Stage 2	12	14.7
Stage 3	67	81.7
Stage 4	3	3.6
***Tumor Location***		
Mid- Rectal	1	1.2
Low – Rectal	81	98.8
***Pre-operative CRM on imaging***		
Involved	74	90.2
Not Involved	8	9.8
***Neo-adjuvant Chemotherapy***		
Yes	79	96.3
No	3	3.7
***Pre-operative Radiation (Duration)***		
Long Corse	78	95.1
Short Course	1	1.2
No Radiation	3	3.6

**Table-II:** Operative and In-Patient Details of Patients.

Variable	Frequency (n=82)	Percentage %
***Mode of Surgery***		
Laparoscopic	81	98.8
Lap converted to Open	1	1.2
***Mode of Perineal Repair***		
Primary repair	64	78
Mesh repair	17	20.7
Gracillis Flap	1	1.2

Pathologically tumor size T was T0:12(14.6%), T2:15(18.92%), T3:50(60.9%), T4:5(6.09%) number of cases. The mean operative time was 340.36±64.51 minutes and the mean blood loss was 99 milliliters. TME was complete in 90.24% (n=74) cases. There was no morality in 90 days. The mean postoperative hospital stay was 6.58±4.64 days. 17(20.8%) cases were pCRM positive and 65 (79.2%) cases were negative. On follow-up, 36(43.9%) cases experienced recurrence (local: 23, distant: 30, combined 17). Twenty-six patients died of disease. One case (1.2%) experienced perineal hernia clinically. Further Post-operative details of patients can be seen in Table-III.

**Table-III:** Post-operative Details of Patients.

Variable	Frequency (n=82)	Percentage %
***Pathological Tumour Size***		
T0	12	14.6
T2	15	18.2
T3	50	61
T4	5	6.1
***Pathological Nodal Status***		
NO	39	47.5
N1	18	22
N2	25	30.4
***Lymphovascular involvement***		
Present	19	23.2
Absent	63	76.8
***Perineuaral Involvement***		
Present	19	23.2
Absent	63	76.8
TME		
Complete	79	96.3
Incomplete	3	3.7
***Tumor Regression Grade***		
Grade 0	12	14.6
Grade 1	13	15.9
Grade 2	31	37.8
Grade 3	23	28
N/A	3	3.7
***Perforation***		
No	74	90.2
Yes	8	9.8
***CRM in mm***		
<1	17	20.7
1	11	13.4
2	5	6.1
>2	36	43.9
CR	13	15.9
***Margins Involved***		
R1	17	20.7
RO	65	79.2
Site R1		
***Anterior+coccyx***	1	1.2
Anterior	9	11
Anterior+Posterior	4	4.9
Anterior+Left Lateral	2	2.4
Posterior	1	1.2
***Adjuvant Treatment***		
Not given	46	56.1
Given	36	43.9
***Sexual dysfunction***		
No	65	79.3
Yes	17	20.7
***Bladder Dysfunction***		
No	74	90.2
Yes	8	9.8
***Perineal Hernia Clinical***		
Yes	1	1.2
No	81	98.8
***Recurrence***		
Yes	36	43.9
No	46	56.1
***Local Recurrence***		
Yes	23	28
No	59	72
***Distant Metastasis***		
Yes	30	36.5
No	52	63.4
***Metastatectomy for distant metastasis***		
Yes	6	20
No	24	80
***Current Status***		
Alive	56	68.3
Dead	26	31.7

## DISCUSSION

In this single center study of 82 patients who underwent ELAPE for locally advanced low rectal carcinoma, the main oncological outcomes included TME which was complete in 90.24 (n=74) cases with 79.2% (n=65) having R0 resections. Despite receiving neoadjuvant chemoradiotherapy, 17 cases were pCRM positive due to persistent disease or disease progression. We believe that tumor biology and young age at presentation along with CRM positivity on final histopathology, plays an important role in the local recurrence and distant metastasis.

Total 44% (n=36) patients developed recurrence including 28% (n=23) local and 36.5% (n=30) distant metastasis compared to 13.2% patients with local recurrence reported by Perdawood et al.^16^ As mentioned earlier tumor biology is one of the main reasons of high recurrence rates in our study because 50% (n=41) of the patients were of poorly differentiated adenocarcinoma. We also observed that out of 36 patients who developed recurrence, 12 patients had signet ring cell morphology which is another possible explanation to this, because 26.8% (n=22) patients’ biology was Signet ring cell which is much higher as compared to the usual observed percentage 1-4% of all rectal cancer.^17^

Further research established that the procedure of ELAPE was the vital cause of attaining perforation rates ranging from 0-7% and these results are comparable to our results of tumor perforations of 9.8% (n-8).^16^,^18^,^19^ Likewise, involved CRM rates were shown to be appreciably decreased by ELAPE in many research in locally advanced rectal carcinoma and are commonly given in a range of 4-14% whereas in our study the CRM was positive in 17 patients (20.7%).^20^-^21^ We believe that our CRM positivity rate is high because 90% (n-74) of our patient’s cohort were CRM-positive before starting neoadjuvant therapy. So basically, these patients did not respond well to neoadjuvant therapy and CRM remained positive. The location of the tumor in all these was low rectal except for one patient of mid rectal and the majority of them were locally advanced.

The mean operative time in our patients was 340.36±64.51 minutes as compared to other results of 300 minutes and the mean blood loss was 99 milliliters similar to other studies of having 92.5 ml.^21^,^22^ The mean postoperative hospital stay was 6.58 days (SD+ 4.64) which is better than 7.5 reported by Zhang et al.^23^ in their results. On the other hand, ELAPE has a number of drawbacks as well, as described by Holm et al.^8^ Firstly, the position of the patient during the perineal part of the surgery needs to be changed to the prone jackknife and this requires extra time. Secondly, there are chances that pelvic nerves and important vessels of the pelvis can be damaged during dissection around levator muscles via the perineal approach and surgeons will enter the pelvic cavity blindly in open procedure. Also, there is always the chance of perineal infections as well.^24^ Lastly, the reconstruction of the pelvic defect is required in some cases. To address these problems, we performed the modified version of ELAPE with the abdominal part being carried out laparoscopically, which reduced the injury to the pelvic structures. Bladder and sexual dysfunction were seen in only 8 and 17 (n=82) cases respectively which is much better than the published data.^21^ Although our results are better than some of the studies but we need to look into these complications separately in detail with a specific scoring system and its comparison to other techniques of surgery as well, similar to what Ahmed J et al and Kim JY et al did in their research.^25^,^26^ But as our aim of the study was to present the main oncological and surgical outcomes of ELAPE, so we did not go into detail about each complication, In the future we are planning to do it.

In recent times, data on ELAPE with a laparoscopic abdominal part has not been presented in our part of the world. So, we presented its outcomes to make it more comprehensive for junior surgeons to understand the technique and important considerations who want to excel in laparoscopic Colorectal Surgery.

### Limitations

First of all, it’s a retrospective study, but data was collected prospectively from HIS. Second, all the consecutive patients with locally advanced rectal cancer patients were included in the study to avoid selection bias. We still believe that further prospectively conducted studies need to be done to see the true benefits of the procedure.

## CONCLUSION

Locally advanced low rectal cancer can be managed safely with an extralevator approach. Laparoscopic procedure makes the anatomical landmarks for levator transection in the pelvic cavity easy and clear for dissection. ELAPE has evolved as a safe procedure for locally advance low rectal cancer.

### Authors’ Contributions:

**NB and IN:** Study lead, study concept and manuscript writing.

**FS:** Conception and design.

**OS and IH:** Data collection and analysis of data.

**SK:** Critical Revision for important intellectual content.

**AAS:** Final approval of the Manuscript.

**NB:** Responsible and accountable for the accuracy of the study.
